# Alcohol Use of German Adults during Different Pandemic Phases: Repeated Cross-Sectional Analyses in the COVID-19 Snapshot Monitoring Study (COSMO)

**DOI:** 10.3390/ijerph19095489

**Published:** 2022-05-01

**Authors:** Melanie Koeger, Hannah Schillok, Stephan Voss, Michaela Coenen, Christina Merkel, Caroline Jung-Sievers, on behalf of the COSMO Study Team

**Affiliations:** 1 Institute for Medical Information Processing, Biometry, and Epidemiology—IBE, Chair of Public Health and Health Services Research, LMU Munich, Elisabeth-Winterhalter-Weg 6, 81377 Munich, Germany; mkoeger@ibe.med.uni-muenchen.de (M.K.); hannah.schillok@ibe.med.uni-muenchen.de (H.S.); svoss@ibe.med.uni-muenchen.de (S.V.); coenen@ibe.med.uni-muenchen.de (M.C.); 2 Pettenkofer School of Public Health, Elisabeth-Winterhalter-Weg 6, 81377 Munich, Germany; 3 Federal Centre for Health Education (BZgA), Maarweg 149-161, 50825 Cologne, Germany; christina.merkel@bzga.de

**Keywords:** COVID-19, alcohol use, lockdown, health behaviours, coping strategies, substance use

## Abstract

There is little evidence on how different COVID-19 pandemic phases influence the alcohol use behaviour of adults. The objective of this study is to investigate alcohol use frequency over different COVID-19 pandemic phases and to identify vulnerable subgroups for risky use behaviour in the German adult population. Survey waves of 14/15 April 2020 (*n* = 1032), 23/24 June 2020 (*n* = 993), and 26/27 January 2021 (*n* = 1001) from the COVID-19 Snapshot Monitoring (COSMO) were analysed. The mean age was 46 ± 15.3 years in April, 46 ± 15.5 years in June, and 45 ± 15.5 years in January. The gender ratio was mostly equal in each survey wave. Descriptive analyses and univariate and multivariate logistic regression analyses for individuals with increased alcohol use frequency (AUF) were performed. 13.2% in April (lockdown), 11.3% in June (easement), and 8.6% in January (lockdown) of participants showed an increased AUF. Individuals with perceived burden, high frustration levels due to protective measures, and young to middle-aged adults were more likely to increase their AUF during different pandemic phases. In conclusion, unfavourable alcohol behaviour might occur as a potentially maladaptive coping strategy in pandemics. Because of potential negative long-term consequences of problematic alcohol use behaviour on health, public health strategies should consider mental health consequences and target addictive behaviour, while also guiding risk groups towards healthy coping strategies such as physical activities during pandemics/crises.

## 1. Introduction

Since the SARS-CoV-2 virus outbreak in Wuhan (China) in December 2019, it has spread all over the world and was declared a global pandemic by the World Health Organization (WHO) on 12 March 2020 [1]. As a result, many countries, including Germany, imposed strict social distancing and domestic quarantine measures to contain the spread of SARS-CoV-2 [2]. Not only did the virus cause severe respiratory infections, but first studies suggested that the pandemic also had an impact on the mental health and substance use behaviour in many individuals [3,4,5]. Previous crises such as the 2008 great recession, the 2001 terrorist attacks on 11 September, and the tsunami in Southeast Asia in 2004 had shown a relationship between an increased alcohol use (AU) and stressors induced by stressful life events, crises, or natural disasters [6,7,8]. In the context of the SARS outbreak in 2003, being exposed to the virus or being quarantined was associated with subsequent alcohol abuse and dependence symptoms among health care workers [9].

There is evidence of a connotation between alcohol use disorders (AUD) and stress [10]. Moreover, constitutive symptoms of depression are related to constitutive motives for AU as a coping strategy [11]. First studies about the mental health of the German population indicated an increase in symptoms of generalized anxiety, depression, psychological distress, and COVID-19-related anxiety during the pandemic [12,13]. Furthermore, loss of control, social isolation, and negative effects on the occupational/financial situation occurred in the German population [14,15]. Therefore, these stressors triggered by the COVID-19 pandemic might have led to maladaptive substance use patterns like an increased alcohol use frequency (AUF) [16]. Increasing the frequency of AU enhances the likelihood that the AUDIT (Alcohol Use Disorders Identification Test) score will rise, increasing the likelihood of heavy drinking or alcohol dependence [17]. Moreover, since alcohol use can impair the immune system, deteriorate mental health issues and the propensity for violent behaviour, and enhance the risk of acute respiratory distress syndrome, AU poses a particular health risk [18,19,20].

With the implementation of lockdowns, the WHO expressed concerns about enhanced AU during social isolation and the onset of AUDs, especially in Europe [21]. Studies by Manthey et al. and Koopmann et al. indicated that this assumption might not be unfounded for the German population in lockdown times [3,15,22]. In a European cross-sectional online survey, 32.7% of German individuals reported an increased AUF (*n* = 1517) [3]. An online survey assessing changes in alcohol and tobacco use behaviour during lockdown with 3245 German adults showed that 35.5% of the individuals consumed more alcohol compared to before the lockdown [22].

Although studies, as mentioned above, have already investigated the effects of crises and the pandemic on substance use behaviour such as alcohol and tobacco use, more surveys are needed to strengthen the empirical evidence and to identify risk groups for maladaptive AU behaviour.

Therefore, the aim of this study was to investigate the AU pattern over different COVID-19 pandemic phases as well as to identify vulnerable subgroups for an increased AUF in the German adult population. Additionally, psychosocial factors such as mental burden, worries, and negative effects induced by the pandemic and pandemic measures were analysed as risk factors concerning unfavourable AU behaviour under this extreme situation. We hypothesised that pandemic-associated stress levels led to an increased AUF during the lockdown in the general German adult population. This could affect vulnerable population groups more than others. In addition, we evaluated various time points as we expected a lower proportion of Germans to increase their AUF in times of easement.

## 2. Materials and Methods

### 2.1. Study Design/Sample

The COVID-19 Snapshot Monitoring (COSMO) study was a joint project of the University of Erfurt (Erfurt, Germany), the Robert Koch-Institute (Berlin, Germany), the Federal Centre for Health Education (Cologne, Germany), the Leibniz Institute for Psychology (Frankfurt, Germany), the Science Media Centre (Cologne, Germany), the Bernhard Nocht Institute for Tropical Medicine (Hamburg GER), and the Yale Institute for Global Health (New Haven, CT, USA) [23]. The aim of the project was to record the mental state and perception of the German population during the COVID-19 pandemic. The project consisted of repeated cross-sectional surveys (waves) conducted at first weekly, later biweekly from 3 March 2020. Approximately 1000 individuals aged 18 to 74 years from the general German population took part in each survey wave.

Study participants were recruited by an ISO 26362:2009-compliant online panel (respondi.de) to represent the distribution of the German population by age, gender, and federal state in terms of the German census at each wave.

Participation was based on voluntariness and anonymity. The study followed the European Data Protection Regulation. Each participant agreed to the terms of the study and provided informed consent before being able to answer the questionnaire. All general information regarding the COSMO study design, population, and recruitment, including inclusion and exclusion criteria and processes are described in the study protocol [23]. Ethical approval was granted by the ethics committee of the University of Erfurt (#20200501).

**Figure 1:**
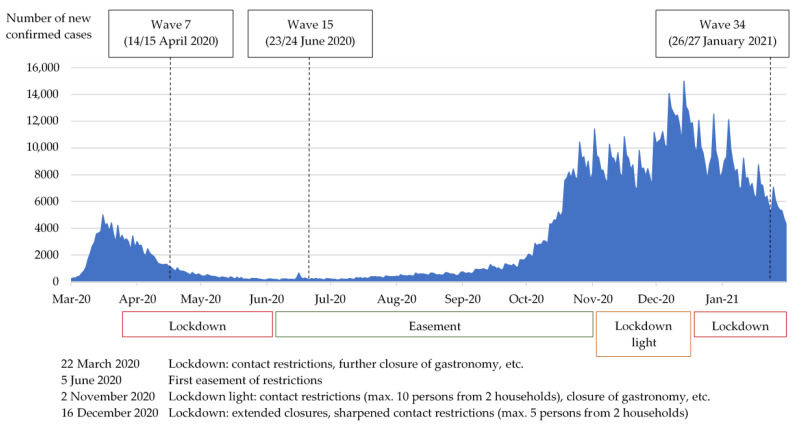
Development of daily incidences (*n*) of SARS-CoV-2 infections in Germany from March 2020 to February 2021. Important changes in pandemic measures for the public are marked. Own illustration based on incidence data from the dashboard of the Robert-Koch Institute https://experience.arcgis.com/experience/478220a4c454480e823b17327b2bf1d4/page/page_1/ (accessed on 28 June 2021).

In this manuscript, the COSMO survey waves of 14/15 April 2020, 23/24 June 2020, and 26/27 January 2021 were analysed. On 14/15 April 2020 (survey wave 7), the German population was in the first so-called lockdown, which was imposed by the German government on 22 March [24,25]. To examine AU behaviour during the period without a lockdown, fewer restrictions, and low incidence values, the survey of 23/24 June 2020 (survey wave 15) was included in the analyses. During the second, larger wave of infections and second strict lockdown in Germany, the ACF was conducted on 26/27 January 2021 (survey wave 34). Thus alcohol behaviour during renewed strict restrictions could be considered (see Figure 1).

### 2.2. Description of Variables and Measures

#### 2.2.1. Demographic Variables

Age was assessed as a continuous variable. For this study, we transformed the data into a categorical variable with the age groups 18–29, 30–44, 45–65, and ≥65 based on the GEDA (German Health Update) study [26]. In addition, gender (i.e., male, female), relationship status (i.e., yes, no), and migration background (i.e., yes, no, I don’t’ know) were used in this report. The respondents were asked in the COSMO survey whether they had children under the age of 18 with several age groups as well as “no” as possible response formats. Response options were collapsed to a binary variable (i.e., yes, no).

#### 2.2.2. Socioeconomic Variables

The net income of the participants was assessed with the income classes < €1250, €1250–1749, €1750–2249, €2250–2999, €3000–3999, €4000–4999, and ≥ €5000 by COSMO. We performed a recoding as follows: (a) <€1250 to capture individuals living near or below the poverty line [27], (b) €1250–2249 to mark the middle-class population including the average income of €1950, (c) €2250–3999 to represent the upper-middle class, and (d) >€4000 to capture the population with a high monthly income [28].

The number of household members (i.e., only me, 2 persons, 3–4 persons, >4 persons, no specification), the status of employment (i.e., yes, no), educational level (<9 years of schooling, >10 years of schooling without A-level, >10 years of schooling with A-level), and federal state of residence (Baden-Württemberg, Bavaria, Berlin, Brandenburg, Bremen, Hamburg, Hesse, Mecklenburg-Western Pomerania, Lower Saxony, North Rhine-Westphalia, Rhineland-Palatinate, Saarland, Saxony, Saxony-Anhalt, Schleswig-Holstein, Thuringia) was assessed by COSMO. For this manuscript, the federal state of residence was recoded into two local regions (i.e., east and west).

#### 2.2.3. Main Outcome: Alcohol Use Frequency

In the COSMO study, AUF was assessed as an indicator for AU behaviour with two variables in the survey waves of 14/15 April 2020, 19/20 May 2020, 23/24 June 2020, 27/28 October 2020, and 26/27 January 2021. The first variable records AUF during the last twelve months. The second refers to AUF during the last four weeks before the survey wave. The study participants were asked how often they drank alcohol such as beer, wine, sparkling wine, spirits, schnapps, cocktails, alcoholic mixed drinks, liqueurs, homemade, or home-distilled alcohol on a weekly basis. Possible answers were “On all days a week“, “On 5 or 6 days a week“, “On 3 or 4 days a week“, “On 2 days a week“, “On 1 day a week“, “On no day”, “I rarely drink alcohol”, and “I never drink alcohol”.

In this work, a new variable was created by comparing the reported AUF during the last four weeks to the AUF during the last twelve months before the survey wave, with the categories “reduced AUF”, “unmodified AUF”, and “increased AUF”. For this purpose, auxiliary variables were formed first in which the answer options “I never drink alcohol”, “I rarely drink alcohol”, and “On no day” were combined since they describe very similar user behaviour. By aggregating the groups “reduced AUF” and “unmodified AUF”, a binary variable was created to analyse the subgroups and factors influencing an increased AUF using univariate and multivariate logistic regression.

The DHS (German Head Office for Addiction Issues) recommends no AU on two days per week as well as a maximum intake of 10–12 g alcohol/day for healthy females and 20–24 g alcohol/day for healthy male adults as low-risk use [29,30]. Adherence to this recommendation was used as an additional outcome measure. For this purpose, we created a binary variable in which the response options “On 5 or 6 days a week” and “On all days of a week” were collapsed, representing no compliance to the recommendation (i.e., AU on ≥5 days/week). The remaining response options were aggregated as reference category “AU on <5 days/week”.

#### 2.2.4. Health-Related Variables

The variable to capture the presence of chronic disease and affiliation to the risk group for SARS-CoV-2 (i.e., yes, no, I don’t know) were conducted by COSMO. For this report, we modelled an item assessing awareness of a personal corona infection with the response formats “yes (current/convalesced)” (originally: yes, confirmed; yes, but not yet confirmed; yes, convalesced), and “no” (originally: no, I don’t know).

To elicit physical activity, subjects were asked how much time they spent on sports, fitness, or physical activity in their free time during a typical week in the current pandemic. The WHO recommends at least 2.5 h of moderate-intensity endurance exercise per week [31]. Based on this recommendation, we created a binary variable that included the categories “<2.5 h/week” and “≥2.5 h/week”.

#### 2.2.5. Variables Assessing Psychosocial Aspects, Reactance to Pandemic Measures and Mental Health

Perceived burden by the pandemic was queried in COSMO (i.e., yes, no). Additionally, study participants were asked how much they worried about (a) losing their job, (b) widening of the gap between rich and poor, (c) getting infected by SARS-CoV-2, and (d) long-term restriction of social life. These questions could be answered on a 7-point Likert scale ranging from very little concern (1) to very much concern (7).

For our analyses, the items were recoded into binary variables describing high levels of worries with response formats of “yes” (5–7) and “no” (1–4). Similarly, the variables assessing the frequency of obtaining information about SARS-CoV-2 (1: never, 7: very often) and frustration due to pandemic measures (1: not at all, 7: very much) were converted to analyse increased information seeking and frustration, respectively (5–7: yes, 1–4: no). Furthermore, two 7-point Likert scale variables about SARS-CoV-2 perception (i.e., the SARS-CoV-2 is something) were conducted by COSMO: One with “I feel helpless about” (1) to “I can actively do something about” (7) and another with “I think a lot about” (1) to “I almost never think about” (7) as response options. For our study, these items were recoded to perceived helplessness regarding SARS-CoV-2 (1–3: yes, 4–7: no) and high levels of rumination about SARS-CoV-2 (1–3: yes, 4–7: no).

### 2.3. Statistical Analysis

The absolute and relative frequencies of all participants and those with an increased AUF were calculated to analyse AUF over different COVID-19 pandemic phases descriptively. For longitudinal comparison, odds ratios (OR), including 95% confidence intervals (CI), were computed by univariate analysis. In addition, proportions of non-drinkers were compared to historical data from the cross-sectional German Health Update (GEDA) study 2014/2015 for age groups and gender using relative frequencies [26]. The GEDA study took place from November 2014 to July 2015 and represented a national health survey. Data was collected by online or paper questionnaire from individuals aged 18 years and older randomly selected from 301 municipalities in Germany.

To assess vulnerable subgroups for an increased AUF in our study, univariate logistic regressions were conducted, and results were reported as OR with corresponding 95% CIs. The influence of COVID-19 associated factors on an increased AUF was analysed respectively. Multivariate logistic regression analysis for an increased AUF was performed, including gender, education, awareness of migration, relationship status, household income, and burden based on previous studies [15,26,32,33,34]. We reported ORs, 95% CIs as well as Nagelkerke’s R^2^. Since COSMO was designed as an explanatory approach, adjustments for multiple testing were not performed. As an additional analysis, a univariate logistic regression for individuals exceeding the DHS recommendation of AU on a maximum of five days a week was estimated [35].

For all logistic regression analyses, determinants with the response options “yes”, “no”, and “I don’t know” (migration background, chronic disease, affiliation to risk group) were collapsed into “no/don’t know” and “yes”. The number of household members (i.e., only me, 2 persons, 3–4 persons, >4 persons, no specification) was recoded to “only me”, “2 persons”, and “≥3 persons” for the statistical analyses. Educational background was collapsed to a binary variable (i.e., no A-level, A-level) due to a small sample size of the category “<9 years of schooling”.

In the case of missing data (household size, net household income, worry employment), these cases were excluded from estimations. To test the significance of variables, *p* values ≤ 0.05 were used (Wald’s test). All analyses were performed using the statistical software R (version 4.0.4) and RStudio (version 1.4.1716) [36].

## 3. Results

### 3.1. Sample Characteristics

In Table 1, study sample characteristics in each survey wave for the total study population and for the subsamples with an increased AUF are presented. The general study population included 1032, 993, and 1001 participants in the survey wave 7 (14/15 April 2020), wave 15 (23/24 June 2020), and wave 34 (26/27 January 2021), respectively. 136 (13.2%), 112 (11.3%), and 86 (8.6%) individuals showed an increased AUF in wave 7 (W7), wave 15 (W15), and wave 34 (W34), respectively. Gender distribution was mostly equal in each wave. The mean age was 46 ± 15.3 years in W7, 46 ± 15.5 years in W15, and 45 ± 15.5 years in W34, with the age group 45 to 64 representing the largest share in the three survey waves. Slightly more than half of the study sample had the highest educational level (A-level). The most common household size was two persons and amounted to approximately 40% in each survey wave. Participants with perceived burden accounted for 40.1% (W7), 36.8% (W15), and 57.1% (W34). For detailed information about general and subsamples, see Table 1.

### 3.2. Alcohol Use Frequency in Different Phases of the Pandemic

Across the different pandemic phases, there was a decrease in the number of individuals who drank alcohol several times a week (W7: 30.2%; W15: 28.7%; W34: 25.6). Daily use was documented by 5.9% (W7) and 4.3% (W34) of study participants, with the lowest proportion at times of easement in W15 amounting to 3.7%.

**Figure 2:**
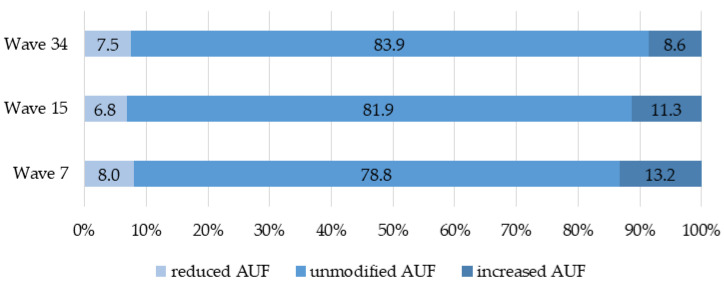
Relative proportions (%) of individuals with reduced, unmodified, and increased AUF in different pandemic phases.

With 13.2% (W7), 11.3% (W15), and 8.6% (W34) of respondents, a decreasing trend across the different pandemic phases is indicated for an increased AUF (see Figure 2). The univariate logistic analysis (see Table S1 in the Supplementary file) showed a significant difference for results between W34 vs. W7 (0.62; 95% CI: 0.47–0.82) and W34 vs. W15 (0.74 [0.55–0.99]). The proportion of subjects with reduced AUF was 8.0% (W7), 6.8% (W15), and 7.5% (W34).

In terms of non-drinkers, the proportions showed marginally higher values for males compared to GEDA data from 2014/2015 (W7: 12.7%, W15: 13.5%, W34: 13.5% vs. 10.3%) [26]. A greater positive deviation from the GEDA data was found for women (W7: 17.0%, W15: 17.8%, W34: 16.4% vs. 13.7%). Further information can be found in Table S2 in the Supplementary file).

### 3.3. Increased Alcohol Use Frequency in Different Subgroups: Univariate Logistic Regression

Table 2 illustrates relative frequencies as well as the corresponding OR with 95% CIs of those who consumed alcohol more frequently during the last four weeks. Men showed an increased AUF more often than women in W7 and W15, though the OR was only significant in W15 (0.65 [0.44–0.97]). With increasing age, the chances of an increased AUF decreased slightly in every survey wave (W7: 0.98 [0.97–0.99]; W15: 0.98 [0.97–1.00]; W34: 0.99 [0.97–1.00]. Especially the age group 18–29 years (W7: 2.35 [1.26–4.37]; W15: 2.47 [CI: 1.06–5.76], W34: 1.41 [0.65–3.02] and 30–44 years (W7: 2.06 [1.13–3.74]; W15: 2.99 [1.37–6.53]; W34: 1.26 [0.61–2.58]) showed higher ORs for an increased AUF. However, the comparator group of those over 65 was relatively small (W7: *n* = 16, W15: *n* = 8, W34: *n* = 11). The odds for an increased AUF were higher for participants in a relationship (significant in W15: 1.73 [1.08–2.75]) or respondents who didn’t live alone. Though, the OR was only significant for the household size of three people in W15 (1.82 [1.06–3.12]) and two people in W34 (2.51 [1.27–4.95]). The odds for an increased AUF were higher among participants with children in W7 and W15, the latter being significant (1.77 [1.18–2.65]). Regarding socioeconomic status, upper-middle class individuals showed the highest odds for an increased AUF with a significant result in W15 (2.15 [1.02–4.52]) and non-significant results in W34 (23.3%). In W15, there was a positive association between increased AUF and employment (1.68 [1.07–2.65]). Chronic illness was negatively related to an increased AUF in W34 (0.55 [0.32–0.92]). More participants with an increased AUF performed the recommended amount of physical activity in W7 (1.57 [1.09–2.25]).

**Figure 3:**
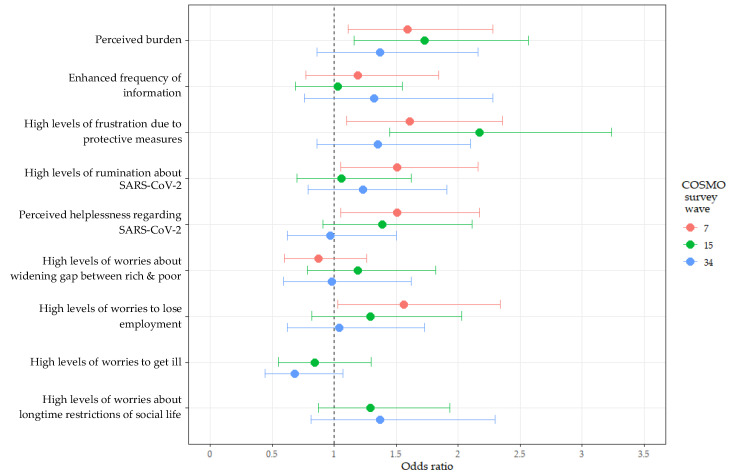
ORs and 95% CIs of univariate analysis for pandemic-related variables of individuals with an increased AUF compared to those with reduced/unmodified AUF (see Table S3 in the Supplementary file).

Considering pandemic-related variables, a positive association between perceived burden and an increased AUF was evident in W7 (1.59 [1.11–2.28]) and W15 (1.73 [1.16–2.57]) (see also Figure 3). High levels of frustration due to pandemic measures were positively associated with an increased AUF in June (1.61 [1.10–2.36]) and April (2.17 [1.45–3.24]). With ORs of 1.51 [1.05–2.16], high levels of rumination about SARS-CoV-2 were positively linked to an increased AUF in W7. A positive association between an increased AUF and perceived helplessness regarding SARS-CoV-2 was observed in W7 (1.51 [1.05–2.17]). Enhanced worries did not show homogeneous patterns of association with an increased AUF: i.e., high levels of worries about getting ill by SARS-CoV-2 and worries about long-time restrictions of social life were not related to an increased AUF, high levels of worries about losing employment were positively associated with an increased AUF in W7 (1.56 [1.03–2.34]).

### 3.4. Multivariate Logistic Regression

Table 3 shows our results of the multivariate logistic regression analysis. Age was positively associated with an increased AUF in W7 (0.98 [0.96–0.99]) and W15 (0.98 [0.97–1.00]), and being in a relationship resulted in a higher OR for an increased AUF in W34 (1.77 [1.01–3.09]). The perceived burden was associated with an increased AUF in W7 (1.53 [1.06–2.20]) and W15 (1.72 [1.14–2.58]) but not in W34.

### 3.5. Adherence to DHS Recommendation for Alcohol Use: Univariate Logistic Regression

Regarding gender, relationship status, household size of two persons, and household net income, the results exceeding the maximum number of days for AC recommended by the DHS were similar to those for an increased AUF. In contrast to the results of the increased AUF analysis, the age groups 18–29 and 30–44 showed low odds of drinking alcohol more than five times per week (see Table S4 in the Supplementary file). With an OR of 1.60 [1.09–2.37] in W7, the perceived burden was positively associated with not complying with the recommendation for AUF, which was comparable to the results for an increased AUF. However, the OR of 1.21 [0.75–1.94] in W15 was not significant, and in W34, it was just below one at 0.95 [0.61–1.51]. Enhanced information frequency was positively related to exceeding the recommendation in W7 (2.08 [1.20–3.61]). All other pandemic-related variables showed an inhomogeneous and non-significant picture across all waves as well as compared to the analysis of an increased AUF. Data is shown in Table S4 in the Supplementary file.

## 4. Discussion

For this work, we analysed COSMO data on AU behaviour at three time points during the COVID-19 pandemic in Germany. An increased AUF was observed during times of lockdown and easement of the pandemic. Taking a public health view, and with regard to possible prevention strategies, we focused on the vulnerable subgroups for an increased AUF in every pandemic phase and how to possibly target them.

Our main results suggest that individuals with perceived burden, high frustration levels due to protective measures, and young to middle-aged adults were more likely to increase AUF over all pandemic phases, regardless of actual context factors. Other factors influencing an increased AUF varied between the different pandemic phases.

First of all, our findings indicate that some increased while some decreased their AUF in different pandemic phases, with slightly more respondents showing an increased AUF. This result was supported by a cross-sectional European online survey with 32.7% increased AUF vs. 24.4% decreased AUF for German individuals (N = 1517) [3]. Furthermore, an online survey of 3245 German adults assessing changes in alcohol use behaviour during the lockdown found a higher proportion of individuals consuming more alcohol than less (35.5% vs. 21.3%) [22]. In other European countries, a higher proportion of an increased AUF was reported for France, Ireland, Poland, and the United Kingdom. In contrast, most European countries had a higher proportion of decreased AUF during the pandemic [3]. The decrease in AUF in Germany was significantly lower compared to other European countries [15]. These results imply that Germany is more vulnerable to an increased AUF than other countries in Europe.

Fewer occasions for AU are given due to the closure of restaurants, bars, and clubs, as well as the cancellation of (large) events in (stricter) lockdown times [37]. Moreover, time restrictions on alcohol sales and night-time curfews might have led to a decreased AUF as well [2]. Since social motives are an important reason for AU, limiting the number of people allowed to meet in private might also have led to a reduction in AUF [16]. However, access to alcohol in supermarkets, gas stations, kiosks, as well as from to-go offerings in restaurants was constant during all pandemic periods with minor restrictions [37]. Additionally, people found new methods to gather for drinking, such as online pub quizzes or online parties [38].

As a second main result, our findings indicated that individuals with perceived burden or high levels of frustration due to protective measures, as well as young to middle-aged adults, were vulnerable to an increased AUF in all pandemic phases. Our multivariate and univariate analysis showed an association between perceived burden and an increased AUF, which was confirmed by another study in Germany in terms of a general increased AU [22]. It has been reported that the German population showed increased symptoms of generalized anxiety, depression, psychological distress, and COVID-19-related fears relatively stable over the different phases of the pandemic [12,13]. As noted in previous studies, these psychological stressors might be important triggers for an increased AUF, implying that alcohol was used as a maladaptive coping strategy [8,33,39]. Moreover, there might be a reciprocal interaction between an increased AUF and heightened levels of perceived burden [40,41]. Recent research suggests that women were more affected by pandemic distress, which is also mainly confirmed by our gender-stratified analysis (data not shown) [13,42]. Nevertheless, AU on ≥5 days/week was still significantly associated with being male in our analysis.

In terms of the temporal dynamics and considering AU as a coping strategy, we expected a lower proportion of an increased AUF in W15 than in W7 and W34 since the pandemic measures were more lenient in W15. Hence, stress levels, and therefore AUF, might have been lower. However, Skoda et al. noted that the high burden levels persisted even when pandemic-induced fears declined and an easement of the pandemic measures was given [13]. On the other hand, there were more occasions for AU in June due to loosened pandemic regulations, and thus, an increased AUF might also have been caused by social drinking motives [16].

High frustration levels due to pandemic measures were positively related to an increased AUF, which can be confirmed by the literature [8,16]. The management of internal emotional stress through AU can be attributed to the inhibitory effect of alcohol on the nervous system [43]. Young adults showed high odds for an increased AUF in our analysis. Studies in the USA and Germany substantiate this result [4,44]. Young adults are in a stage of life where crucial decisions for their future have to be made, e.g., regarding career and relationships [45]. Phases of lockdown and social stagnation might have restricted them in these processes, which can increase the burden and thus AUF [46]. In addition, they are particularly reliant on marginal employment such as waitressing to make a living, which was lost due to the pandemic measures. This might have led to economic difficulties and potential risk factors for an increased AU [34]. As the early onset of AU is related to an enhanced risk of alcohol abuse and dependence, there might be a higher prevalence of AUD in the future [47]. However, Manthey et al. reported that decreased AU was mainly found in younger German individuals [15]. Additionally, recent studies indicated that young adults were more likely to experience not only an increase but also a decrease in AU during the pandemic [44,48]. Therefore, further research is needed to investigate the AU behaviour of young adults during a pandemic.

The association between being in a relationship and an increased AUF (W15) could be potentially explained by increased peer pressure, relationship problems, or a maladaptive coping strategy of boredom and/or stress.

Consistent with the existing literature, our work also identified middle-aged individuals as a vulnerable subgroup for an increased AUF [22,49]. Our work and a recent study have shown an association between parenting, which is often linked to middle age, and an increased AUF [50]. Furthermore, middle-aged individuals might be most at risk of financial dependence, as they are likely to have only been employed for a few years, have to provide for a family, and might have a loan to repay [49].

Besides, our analysis implied that individuals with perceived helplessness and high levels of rumination were at risk for an increased AUF in W7. Wolitzky-Taylor et al. reported a link between ruminative thinking and AUD [51]. Since worries are related to burden, we assumed that analyses of the effects of worries on AUF would have yielded a higher degree of correlation. However, an increase in worries showed different probabilities of an increased AUF, which might be explained by various personality traits [34].

Regarding socioeconomic status (income, employment, migration background, and education), our analyses showed high variability. An underrepresentation of individuals with low socioeconomic status in this snapshot survey might have led to distorted results in our study. Nevertheless, previous research has reviewed that unemployment and financial stressors are positively and negatively associated with adverse AU behaviour, respectively [8,34].

Interestingly, performing the recommended amount of physical activity was positively associated with an increased AUF in W7 (1.57 [1.09–2.25]). This could imply that individuals also engage in good coping strategies besides maladaptive ones [52]. Further research is needed to clarify the relationship between physical activity and AU behaviour and possible mediators.

As there is no safe level of AU and a previous SARS outbreak has shown the occurrence of later alcohol abuse and dependence symptoms, it is important to inform the population about the risks of AU/an increased AUF [9,10,18]. Besides addictive concerns, social dangers can also arise from adverse alcohol behaviour, e.g., domestic violence, accidents, and crime [18,19,53]. Regarding future pandemics, more stringent public health strategies aimed at limiting AU/access to alcoholic beverages are needed (i.e., stricter time restrictions on the sale of alcohol in retail outlets, restrictions on alcohol marketing, or higher taxes/prices on alcoholic beverages) [53,54]. Additionally, more information about maladaptive coping strategies and their negative impact on health should be made available to the general population (e.g., easily accessible online fact sheets and various digital apps). Regarding burden as a risk factor for maladaptive alcohol behaviour, more psychosocial supports and services should be offered in times of pandemics or crises. Additionally, incentives for physical activity and other healthy coping strategies should be created by policymakers, especially for young to middle-aged adults during pandemics.

### Strengths and Limitations

In terms of strengths, this repeated cross-sectional study design allows good insights into different pandemic phases. With about 1000 participants in each survey wave, a sufficient study population is given. Gender and age distribution are representative due to COSMO study recruitment. However, small sub-samples for given sub-questions might have distorted results in some sub-analyses.

Still, study limitations also need to be considered. Due to the repeated cross-sectional study design, no conclusions can be drawn about the causality of the results. Although a cross-sectional design was chosen for this snapshot study, a prospective longitudinal study with the same individuals would have provided better insights into the trajectories of individual AU behaviour in relation to changing contextual factors. Considering bias, recall bias and social desirability bias might have occurred since COSMO collects self-reported data. In our sample, W34 was conducted nine months after the onset of the pandemic. Hence, the results of W34 might be distorted due to recall bias, habituation effect, or holidays that ended between 2 and 10 January 2021, respectively, to the federal state. In addition, pre-pandemic data on individual AU behaviour was unavailable in this study design.

It should be noted that the variables of COSMO applied to survey AU are not validated instruments and only assess the frequency of AU. Valid statements about hazardous AU or alcohol dependency can only be made by assessing the consumed quantity, heavy drinking, and items assessing dependence symptoms [17]. Nevertheless, frequency represents an easier variable to remember than the exact amount consumed. Regarding DHS recommendation, using five days a week would still be acceptable. However, since use five days a week was assessed in a category together with six days a week, we had to consider five days a week as exceeding the recommendation. After all, some contextual factors that could be determinants for an increased (e.g., job loss, boredom, or quarantine) or decreased (e.g., earlier closing times for on-use outlets or alcohol restrictions in public areas) AUF during the COVID-19 pandemic were not collected by the COSMO survey. Due to the snapshot design of the study, no profound conclusions can be drawn for individual topics.

## 5. Conclusions

In conclusion, an increased AUF was present over different phases of the COVID-19 pandemic. Individuals showing perceived burden, high levels of frustration due to protective measures, and young to middle-aged adults were identified as most vulnerable to an increased AUF. Because of the potential negative long-term consequences on health, public health strategies should target addictive behaviour during pandemics/crises while considering the aforementioned vulnerable groups. In addition, general preventive measures such as access to alcoholic beverages should be more strictly limited. The public should be guided towards healthy coping strategies such as physical activities instead of AU. Moreover, further research is needed to examine the extent and the motives for an increased AUF, binge drinking, and heavy drinking, as well as the impact of the COVID-19 pandemic on future AU behaviour and AUDs.

## Figures and Tables

**Table 1:** Sample characteristics.

Characteristics	Wave 7 (14/15 April 2020)				Wave 15 (23/24 June 2020)				Wave 34 (26/27 January 2021)			
	Total		Increased AUF		Total		Increased AUF		Total		Increased AUF	
	*n*/mean	%/SD	*n*/mean	%/SD	*n*/mean	%/SD	*n*/mean	%/SD	*n*/mean	%/SD	*n*/mean	%/SD
Total	1032	-	136	13.2	993	-	112	11.3	1001	-	86	8.6
Gender												
Male	503	48.7	75	55.1	483	48.6	65	58.0	504	50.3	44	51.2
Female	529	51.3	61	44.9	510	51.4	47	42.0	497	49.7	42	48.8
Age (continuous)	46	15.7	41	15.3	46	15.5	43	14.3	45	15.5	41	15.7
Age group												
18–29	207	20.1	39	28.7	178	17.9	21	18.8	192	19.2	21	24.4
30–44	296	28.7	50	36.8	309	31.1	43	38.4	313	31.3	31	36.0
45–64	351	34.0	31	22.8	350	35.2	40	35.7	359	35.9	23	26.7
≥65	178	17.2	16	11.8	156	15.7	8	7.1	137	13.7	11	12.8
Education level												
<9 years	127	12.3	13	10.2	112	11.3	11	9.8	119	11.9	9	7.6
>10 years (no A-level)	326	31.6	41	12.6	340	34.2	45	13.2	309	30.9	23	7.4
>10 years (A-level)	579	56.1	82	14.2	541	54.5	56	10.4	573	57.2	54	9.4
Migration background												
No	894	86.6	119	12.0	839	84.5	88	16.0	811	81.0	69	9.1
Yes	133	12.9	16	13.3	150	15.1	24	1.5	187	18.7	17	8.5
I don’t know	5	0.5	1	20.0	4	0.4	0	0	3	0.3	0	0.0
Local region												
East	168	16.3	19	14.0	165	16.6	17	15.2	161	16.1	11	12.8
West	864	83.7	117	86.0	828	83.4	95	84.8	840	83.9	75	87.2
Relationship												
No	316	30.6	41	30.1	317	31.9	25	22.3	309	30.9	20	23.3
Yes	716	69.4	95	69.9	676	68.1	87	77.7	692	69.1	66	76.7
Children												
No Children	725	70.3	88	64.7	698	70.3	66	58.9	716	71.5	62	72.1
Children	307	29.7	48	35.3	295	29.7	46	41.1	285	28.5	24	27.9
Household size												
Just me	236	22.9	29	12.3	262	26.4	21	8.0	231	23.1	11	5.6
2 persons	441	42.7	53	12.0	387	39.0	44	11.4	404	40.4	45	11.1
3–4 persons	308	29.8	50	16.2	293	29.5	37	12.6	302	30.2	25	6.3
>4 persons	47	4.6	4	8.5	51	5.1	10	19.6	61	6.1	5	8.2
No specification	0	0	0	0	0	0	0	0.0	3	0.3	0	0.0
Household net income												
<€1250	NA	NA	NA	NA	142	14.3	11	9.8	115	11.5	10	11.6
€1250–2249	NA	NA	NA	NA	249	25.1	24	21.4	245	24.5	21	24.4
€2250–3999	NA	NA	NA	NA	352	35.4	47	42.0	367	36.7	27	31.4
>€4000	NA	NA	NA	NA	170	17.1	26	23.2	199	19.9	20	23.3
No specification	NA	NA	NA	NA	80	8.1	4	3.6	75	7.5	8	9.3
Employment												
No	NA	NA	NA	NA	334	33.6	27	24.1	309	30.9	28	32.6
Yes	NA	NA	NA	NA	659	66.4	85	75.9	692	69.1	58	67.4
Chronic disease												
No	641	62.1	92	10.7	632	63.6	71	10.9	634	63.3	64	8.0
Yes	345	33.4	37	14.4	338	34.0	37	11.2	332	33.2	19	9.0
I don’t know	46	4.5	7	15.2	23	2.3	4	17.4	35	3.5	3	2.9
Personal infection with SARS-CoV-2												
No	1013	98.2	99	72.8	966	97.3	109	97.3	945	94.4	80	93.0
Yes (current/convalesced)	19	1.8	37	27.2	27	2.7	3	2.7	56	5.6	6	7.0
Affiliation to risk group for SARS-CoV-2												
No	NA	NA	NA	NA	475	47.8	64	9.3	572	57.1	57	6.1
Yes	NA	NA	NA	NA	518	52.2	48	13.5	349	34.9	21	11.2
Don’t know	NA	NA	NA	NA	0	0	0	0	80	8	8	10.0
Perceived burden												
No	618	59.9	68	50.0	639	64.4	59	52.7	429	42.9	31	36.0
Yes	414	40.1	68	50.0	354	35.6	53	47.3	572	57.1	55	64.0
AUF during the last four weeks												
Never	175	17.0	0	0.0	177	17.8	0	0.0	164	16.4	0	0.0
Rarely	319	30.9	0	0.0	332	33.4	0	0.0	371	37.1	0	0.0
Once a week	165	16.0	39	28.7	162	16.3	34	30.4	167	16.7	28	32.6
Several times per week	312	30.2	86	63.2	285	28.7	74	66.1	256	25.6	51	59.3
On all days of the week	61	5.9	11	8.1	37	3.7	4	3.6	43	4.3	7	8.1

Abbreviations: AUF: alcohol use frequency, N: number of cases in the total sample, *n*: number of cases in the subsamples with an increased AUF, SD: standard deviation, NA: not available (not collected in this wave).

**Table 2:** Univariate analysis: relative frequencies, ORs, and 95% CIs of individuals with an increased AUF compared to those with reduced/unmodified AUF between different subgroups.

Characteristics	Wave 7 (14/15 April 2020)			Wave 15 (23/24 June 2020)			Wave 34 (26/27 January 2021)		
	Increased		95% CI	Increased		95% CI	Increased		95% CI
	AUF (%)	OR		AUF (%)	OR		AUF (%)	OR	
Gender									
Male (reference)	14.9			13.5			8.7		
Female	11.5	0.74	[0.52–1.07]	9.2	**0.65**	[0.44–0.97] *	8.5	0.97	[0.62–1.50]
Age (continuous)		**0.98**	[0.97–0.99] ***		**0.98**	[0.97–1.00] *		0.99	[0.97–1.00]
Age group									
≥65 (reference)	9.0			5.1			8.0		
18–29	18.8	**2.35**	[1.26–4.37] **	11.8	**2.47**	[1.06–5.76] *	10.9	1.41	[0.65–3.02]
30–44	16.9	**2.06**	[1.13–3.74] *	13.9	**2.99**	[1.37–6.53] **	9.9	1.26	[0.61–2.58]
45–64	8.8	0.98	[0.52–1.85]	11.4	**2.39**	[1.09–5.23] *	6.4	0.78	[0.37–1.66]
Educational level									
No A-Level (reference)	11.9			12.4			7.5		
A-Level	14.2	1.22	[0.84–1.76]	10.4	0.82	[0.55–1.21]	9.4	1.29	[0.82–2.03]
Migration background									
No/Don’t know (reference)	13.3			10.4			8.5		
Yes	12.0	0.89	[0.51–1.55]	16.0	**1.63**	[1.00–2.65] *	9.1	1.08	[0.62–1.88]
Local region									
East	11.3			10.3			6.8		
West	13.5	1.23	[0.73–2.06]	11.5	1.13	[0.65–1.95]	8.9	1.34	[0.69–2.58]
Relationship									
No (reference)	13.0			7.9			6.5		
Yes	13.3	1.03	[0.69–1.52]	12.9	**1.73**	[1.08–2.75] *	9.5	1.52	[0.91–2.56]
Children									
No (reference)	12.1			9.5			8.7		
Yes	15.6	1.34	[0.92–1.96]	15.6	**1.77**	[1.18–2.65] **	8.4	0.97	[0.59–1.59]
Household size									
Just me (reference)	12.3			8.0			4.8		
2 people	12.0	0.98	[0.60–1.58]	11.4	1.47	[0.85–2.54]	11.1	**2.51**	[1.27–4.95] **
≥3 people	15.2	1.38	[0.85–2.26]	13.7	**1.82**	[1.06–3.12] *	8.3	1.81	[0.87–3.75]
Household net income									
<€1250 (reference)	NA	NA	NA	7.7			8.7		
€1250–2249	NA	NA	NA	9.6	1.27	[0.60–2.68]	8.6	0.98	[0.45–2.16]
€2250–3999	NA	NA	NA	13.4	1.84	[0.92–3.65]	7.4	0.83	[0.39–1.78]
>€4000	NA	NA	NA	15.3	**2.15**	[1.02–4.52] *	10.1	1.17	[0.53–2.60]
No specification	NA	NA	NA	5.0	0.63	[0.19–2.04]	10.7	1.25	[0.47–3.34]
Employment									
No (reference)	NA	NA	NA	8.1			9.1		
Yes	NA	NA	NA	12.9	**1.68**	[1.07–2.65] *	8.4	0.92	[0.57–1.47]
Chronic disease									
No/Don’t know (reference)	14.4			11.5			10.0		
Yes	10.7	0.71	[0.48–1.07]	10.9	0.97	[0.64–1.48]	5.7	**0.55**	[0.32–0.92] *
Physical activity									
<2.5 h/week (reference)	11.0			10.8			NA	NA	NA
≥2.5 h/week	16.2	**1.57**	[1.09–2.25] **	12.1	1.13	[0.75–1.69]	NA	NA	NA

To view the *n* of the subgroups, refer to Table 1. * *p* < 0.05; ** *p* < 0.01, *** *p* < 0.001; marked in bold. Abbreviations: AUF: alcohol use frequency, OR: odds ratio, CI: confidence interval, NA: not available (not collected in this wave). Non-respondents for household size were not included in the analysis.

**Table 3:** Multivariate analysis: adjusted ORs and 95% CIs of individuals with an increased AUF compared to those with reduced/unmodified AUF between different subgroups.

Characteristics	Wave 7 (14/15 April 2020)		Wave 15 (23/24 June 2020)		Wave 34 (26/27 January 2021)	
	OR	95% CI	OR	95% CI	OR	95% CI
Gender						
Male (reference)						
Female	0.69	[0.48–1.00]	0.62	[0.42–1.04]	0.95	[0.60–1.48]
Age (continuous)	**0.98**	[0.96–0.99] ***	**0.98**	[0.97–1.00] *	0.99	[0.97–1.00]
Educational level						
No A-Level (reference)						
A-Level	0.98	[0.66–1.45]	0.68	[0.45–1.04]	1.22	[0.75–1.99]
Migration background						
No/Don’t know (reference)						
Yes	0.73	[0.41–1.30]	1.52	[0.91–2.53]	0.98	[0.55–1.73]
Relationship						
No (reference)						
Yes	1.11	[0.75–1.66]	1.50	[0.89–2.52]	**1.77**	[1.01–3.09] *
Household net income						
<€1250 (reference)	NA	NA				
€1250–2249	NA	NA	1.16	[0.54–2.48]	0.91	[0.41–2.03]
€2250–3999	NA	NA	1.63	[0.78–3.40]	0.64	[0.29–1.43]
>€4000	NA	NA	1.93	[0.85–4.36]	0.87	[0.37–2.03]
No specification	NA	NA	0.56	[0.17–1.88]	1.08	[0.40–2.93]
Perceived burden						
No (reference)						
Yes	**1.53**	[1.06–2.20] *	**1.72**	[1.14–2.58] *	1.33	[0.83–2.13]
Nagelkerke Pseudo R^2^	0.0434		0.0707		0.0251	

To view the *n* of the subgroups, refer to Table 1. * *p* < 0.05; ** *p* < 0.01, *** *p* < 0.001; marked in bold. Abbreviations: AUF: alcohol use frequency, OR: odds ratio, CI: confidence interval, NA: not available (not collected in this wave).

## Data Availability

Data are not publicly available, but interested parties may contact the authors for more information. The data are not publicly available due to ethical restrictions.

## Supplementary Materials

The following supporting information can be downloaded at: https://www.mdpi.com/article/10.3390/ijerph19095489/s1, Supplementary Table S1. Univariate analysis for modification of AUF (last four weeks vs. last 12 months) between different pandemic phases: ORs and 95% CIs. Supplementary Table S2. Comparison of relative proportions of non-drinkers during the pandemic (AUF in the last four weeks: category “never”) to pre-pandemic data (GEDA 14/15). Supplementary Table S3. Univariate analysis for pandemic-related variables: relative frequencies, ORs, and 95% CIs of individuals with increased AUF compared to those with reduced/unmodified AUF between different subgroups. Supplementary Table S4. Univariate analysis: relative frequencies, ORs, and 95% CIs of individuals exceeding (AU on ≥5 days/week) compared with those who adhere to the DHS recommendation (AU on <5 days/week).
