# Supplementary material for: Alcohol Use of German Adults during Different Pandemic Phases: Repeated Cross-Sectional Analyses in the COVID-19 Snapshot Monitoring Study (COSMO)
Source: Int J Environ Res Public Health. 2022 May 1;19(9):5489. doi: 10.3390/ijerph19095489 (PMC9099585; doi:10.3390/ijerph19095489)
Supplement: Supplementary file 1 [file ijerph-19-05489-s001.zip › ijerph-1551674-supplementary.pdf]

International Journal of
*Environmental Research and Public Health*

MDPI

## Modification of Alcohol Consumption Frequency

**Supplementary Table S1.** Univariate analysis for modification of AUF (last four weeks vs. last 12 months) between different pandemic phases: ORs and 95% CIs.

| Characteristics | Wave 15 vs. 7 | | Wave 34 vs. 7 | | Wave 34 vs.15 | |
|---|---|---|---|---|---|---|
| | OR | 95 % CI | OR | 95 % CI | OR | 95 % CI |
| Reduced AUF | 0.84 | [0.60–1.17] | 0.93 | [0.67–1.28] | 1.10 | [0.78–1.55] |
| Unmodified AUF | 1.22 | [0.98–1.52] | **1.41** | **[1.12–1.76] **** | 1.16 | [0.91–1.46] |
| Increased AUF | 0.84 | [0.64–1.09] | **0.62** | **[0.47–0.82] ***** | **0.74** | **[0.55–0.99] *** |

* $p < 0.05$; ** $p < 0.01$, *** $p < 0.001$; marked in bold. Abbreviations: AUF: alcohol consumption frequency, OR: odds ratio, CI: confidence interval.

## Pre-pandemic to Pandemic Comparison of Non-Drinkers

**Supplementary Table S2.** Comparison of relative proportions of non-drinkers during the pandemic (AUF in the last four weeks: category "never") to pre-pandemic data (GEDA 14/15) [26].

| Characteristics | GEDA 14/15 | Wave 7 (14/15 April 2020) | | Wave 15 (23/24 June 2020) | | Wave 34 (26/27 January 2021) | |
|---|---|---|---|---|---|---|---|
| | % | *n* | % | *n* | % | *n* | % |
| Men (total) | 10.3 | 64 | 12.7 | 65 | 13.5 | 68 | 13.5 |
| 18–29 | 10.5 | 13 | 13.4 | 13 | 15.7 | 8 | 8.2 |
| 30–44 | 9.4 | 14 | 11.3 | 13 | 8.7 | 15 | 9.6 |
| 45–64 | 10.0 | 27 | 14.4 | 28 | 15.7 | 34 | 19.1 |
| ≥65 | 11.8 | 10 | 10.5 | 11 | 15.1 | 11 | 15.2 |
| Women (total) | 16.9 | 111 | 21.0 | 112 | 22.0 | 96 | 19.3 |
| 18–29 | 15.0 | 22 | 20.0 | 22 | 23.2 | 21 | 22.1 |
| 30–44 | 16.5 | 30 | 17.4 | 30 | 18.8 | 38 | 24.4 |
| 45–64 | 12.5 | 39 | 23.8 | 40 | 23.3 | 28 | 15.5 |
| ≥65 | 24.3 | 20 | 24.1 | 20 | 24.1 | 9 | 13.8 |
| Total (men and women) | 13.7 | 175 | 17.0 | 177 | 17.8 | 164 | 16.4 |

## Univariate Analysis—Pandemic-Related Variables

**Supplementary Table S3.** Univariate analysis for pandemic-related variables: relative frequencies, ORs, and 95% CIs of individuals with increased AUF compared to those with reduced/unmodified AUF between different subgroups.

| Characteristics | Wave 7 (14/15 April 2020) | | | Wave 15 (23/24 June 2020) | | | Wave 34 (26/27 January 2021) | | |
|---|---|---|---|---|---|---|---|---|---|
| | Increased AUF (%) | OR | 95% CI | Increased AUF (%) | OR | 95% CI | Increased AUF (%) | OR | 95% CI |
| Perceived burden | | | | | | | | | |
| No (reference) | 11.0 | | | 9.2 | | | 7.2 | | |
| Yes | 16.4 | **1.59** | **[1.11–2.28] *** | 15.0 | **1.73** | **[1.16–2.57] **** | 9.6 | 1.37 | [0.86–2.16] |
| Enhanced frequency of information | | | | | | | | | |
| No | 11.7 | | | 11.1 | | | 7.1 | | |
| Yes | 13.6 | 1.19 | [0.77–1.84] | 11.4 | 1.03 | [0.69–1.55] | 9.1 | 1.32 | [0.76–2.28] |
| High levels of frustration due to protective measures | | | | | | | | | |
| No (reference) | 11.6 | | | 8.8 | | | 7.6 | | |
| Yes | 17.4 | **1.61** | **[1.10–2.36] *** | 17.3 | **2.17** | **[1.45–3.24] ***** | 9.9 | 1.35 | [0.86–2.10] |
| Perceived helplessness regarding SARS-CoV-2 | | | | | | | | | |
| No (reference) | 11.3 | | | 11.1 | | | 7.9 | | |
| Yes | 16.1 | **1.51** | **[1.05–2.16] *** | 11.7 | 1.06 | [0.70–1.62] | 9.6 | 1.23 | [0.79–1.91] |
| High levels of rumination about SARS-CoV-2 | | | | | | | | | |
| No (reference) | 11.1 | | | 10.3 | | | 8.7 | | |
| Yes | 16.1 | **1.51** | **[1.05–2.17] *** | 13.8 | 1.39 | [0.91–2.11] | 8.4 | 0.97 | [0.62–1.50] |

| | | | | | | | | | |
|---|---|---|---|---|---|---|---|---|---|
| **High levels of worries…** | | | | | | | | | |
| … about widening gap between rich and poor | | | | | | | | | |
| No (reference) | 14.2 | | | 10.1 | | | 8.7 | | |
| Yes | 12.6 | 0.87 | [0.60–1.26] | 11.9 | 1.19 | [0.78–1.82] | 8.5 | 0.98 | [0.59–1.62] |
| ... to lose employment | | | | | | | | | |
| No (reference) | 11.8 | | | 11.0 | | | 8.1 | | |
| Yes | 17.2 | **1.56** | **[1.03–2.34] *** | 13.8 | 1.29 | [0.82–2.03] | 8.4 | 1.04 | [0.62–1.73] |
| … to get ill | | | | | | | | | |
| No (reference) | NA | NA | NA | 11.8 | | | 10.0 | | |
| Yes | NA | NA | NA | 10.1 | 0.84 | [0.55–1.30] | 7.1 | 0.68 | [0.44–1.07] |
| … about longtime restrictions of social life | | | | | | | | | |
| No (reference) | NA | NA | NA | 10.0 | | | 6.9 | | |
| Yes | NA | NA | NA | 12.5 | 1.29 | [0.87–1.93] | 9.3 | 1.37 | [0.81–2.30] |

To view the *n* of the subgroups, refer to Table 1. * $p < 0.05$; ** $p < 0.01$, *** $p < 0.001$; marked in bold. Abbreviations: AUF: alcohol consumption frequency, OR: odds ratio, CI: confidence interval, NA: not available (not collected in this wave). Non respondents for high levels of worries to lose employment were not included in the analysis.

## Additional Analysis—Exceeding DHS Recommendation

**SupplementaryTable S4.** Univariate analysis: relative frequencies, ORs, and 95% CIs of individuals exceeding (AU on ≥5 days/week) compared with those who adhere to the DHS recommendation (AU on <5 days/week).

| **Characteristics** | **Wave 7 (14/15 April 2020)** | | | **Wave 15 (23/24 June 2020)** | | | **Wave 34 (26/27 January 2021)** | | |
|---|---|---|---|---|---|---|---|---|---|
| | **AU on ≥5 days/week (%)** | **OR** | **95% CI** | **AU on ≥5 days/week (%)** | **OR** | **95% CI** | **AU on ≥5 days/week (%)** | **OR** | **95% CI** |
| Gender | | | | | | | | | |
| Male (reference) | 15.9 | | | 11.6 | | | 11.7 | | |
| Female | 6.6 | **0.37** | **[0.25–0.57] ***** | 4.3 | **0.34** | **[0.21–0.57] ***** | 4.6 | 0.37 | **[0.22–0.60] ***** |
| Age (continuous) | | **1.03** | **[1.02–1.04] ***** | | **1.03** | **[1.01–1.05] ***** | | 1.03 | **[1.01–1.04] ***** |
| Age group | | | | | | | | | |
| ≥65 (reference) | 15.7 | | | 14.1 | | | 13.1 | | |
| 18–29 | 6.3 | **0.36** | **[0.18–0.72] **** | 3.4 | **0.21** | **[0.08–0.54] **** | 4.2 | **0.29** | **[0.12–0.68] **** |
| 30–44 | 7.4 | **0.43** | **[0.24–0.78] **** | 5.2 | **0.33** | **[0.17–0.65] **** | 7.3 | 0.52 | [0.27–1.01] |
| 45–64 | 14.8 | 0.93 | [0.57–1.54] | 9.7 | 0.66 | [0.37–1.16] | 9.2 | 0.67 | [0.36–1.23] |
| Educational level | | | | | | | | | |
| No A-Level (reference) | 10.6 | | | 8.6 | | | 9.1 | | |
| A-Level | 11.6 | 1.10 | [0.75–1.64] | 7.2 | 0.82 | [0.52–1.31] | 7.5 | 0.81 | [0.51–1.27] |
| Migration background | | | | | | | | | |
| No/Don't know (reference) | 11.7 | | | 7.8 | | | 8.8 | | |
| Yes | 7.5 | 0.61 | [0.31–1.21] | 8.0 | 1.02 | [0.51–1.94] | 5.3 | 0.58 | [0.29–1.15] |
| Local region | | | | | | | | | |
| East | 8.9 | | | 8.5 | | | 9.3 | | |
| West | 11.6 | 1.34 | [0.76–2.36] | 7.7 | 0.9 | [0.49–1.65] | 8.0 | 0.84 | [0.47–1.52] |
| Relationship status | | | | | | | | | |
| No (reference) | 10.1 | | | 6.9 | | | 5.2 | | |
| Yes | 11.6 | 1.16 | [0.76–1.79] | 8.3 | 1.21 | [0.73–2.02] | 9.5 | **1.93** | **[1.10–3.39] *** |
| Children | | | | | | | | | |
| No (reference) | 11.2 | | | 8.5 | | | 8.8 | | |
| Yes | 11.1 | 0.99 | [0.65–1.51] | 6.4 | 0.75 | [0.44–1.27] | 6.7 | 0.74 | [0.43–1.26] |
| Household size | | | | | | | | | |
| Just me (reference) | 12.3 | | | 8.4 | | | 5.6 | | |
| 2 people | 12.2 | 0.996 | [0.62–1.61] | 9.6 | 1.15 | [0.66–2.00] | 11.1 | **2.10** | **[1.11–3.99] *** |
| ≥3 people | 9.0 | 0.77 | [0.45–1.32] | 5.5 | 0.64 | [0.34–1.20] | 6.3 | 1.13 | [0.54–2.33] |
| Local region | | | | | | | | | |
| East | | | | | | | 9.3 | | |
| West | | | | | | | 8.0 | | |
| Household net income | | | | | | | | | |
| <€1250 (reference) | NA | NA | NA | 7.0 | | | 3.5 | | |

| | | | | | | | | | |
|---|---|---|---|---|---|---|---|---|---|
| € 1250–2249 | NA | NA | NA | 7.2 | 1.03 | [0.46–2.29] | 8.2 | 2.47 | [0.82–7.39] |
| € 2250–3999 | NA | NA | NA | 9.1 | 1.32 | [0.63–2.76] | 9.3 | 2.83 | [0.98–8.16] |
| >€4000 | NA | NA | NA | 8.2 | 1.18 | [0.51–2.75] | 10.6 | **3.27** | **[1.10–9.79]** * |
| No specification | NA | NA | NA | 5.0 | 0.69 | [0.21–2.29] | 4.0 | 1.16 | [0.25–5.32] |
| Employment | | | | | | | | | |
| No (reference) | NA | NA | NA | 9.0 | | | 9.1 | | |
| Yes | NA | NA | NA | 7.3 | 0.8 | [0.49–1.28] | 7.8 | 0.85 | [0.53–1.37] |
| Chronic disease | | | | | | | | | |
| No/Don't know (reference) | 10.5 | | | 6.9 | | | 7.8 | | |
| Yes | 12.5 | 1.22 | [0.81–1.82] | 9.8 | 1.47 | [0.92–2.35] | 9.0 | 1.18 | [0.74–1.89] |
| Physical activity | | | | | | | | | |
| <2.5 hours/week (reference) | 11.1 | | | 7.5 | | | NA | NA | NA |
| ≥2.5 hours/week | 11.2 | 1.00 | [0.68–1.48] | 8.5 | 1.15 | [0.71–1.84] | NA | NA | NA |
| Perceived burden | | | | | | | | | |
| No (reference) | 9.2 | | | 7.4 | | | 8.4 | | |
| Yes | 14.0 | **1.60** | **[1.09–2.37]** * | 8.5 | 1.21 | [0.75–1.94] | 8.0 | 0.95 | [0.61–1.51] |
| Enhanced frequency of information | | | | | | | | | |
| No (reference) | 6.5 | | | 9.3 | | | 8.7 | | |
| Yes | 12.6 | **2.08** | **[1.20–3.61]** * | 6.9 | 0.72 | [0.45–1.14] | 8.0 | 0.91 | [0.54–1.54] |
| High levels of frustration due to protective measures | | | | | | | | | |
| No | 6.5 | | | 7.0 | | | 8.3 | | |
| Yes | 12.6 | 1.50 | [0.99–2.26] | 10.0 | 1.49 | [0.92–2.41] | 8.1 | 0.97 | [0.62–1.54] |
| Perceived helplessness regarding SARS-CoV-2 | | | | | | | | | |
| No | 10 | | | 8.6 | | | 8.6 | | |
| Yes | 14.2 | 0.97 | [0.65–1.44] | 6.3 | 0.72 | [0.43–1.22] | 7.6 | 0.87 | [0.54–1.39] |
| High levels of rumination about SARS-CoV-2 | | | | | | | | | |
| No | 11.3 | | | 7.9 | | | 8.7 | | |
| Yes | 10.9 | 1.13 | [0.77–1.67] | 7.6 | 0.95 | [0.57–1.61] | 7.6 | 0.86 | [0.54–1.35] |
| **High levels of worries…** | | | | | | | | | |
| … about widening gap between rich and poor | | | | | | | | | |
| No | 10.6 | | | 6.3 | | | 9.1 | | |
| Yes | 11.9 | 1.22 | [0.81–1.84] | 8.7 | 1.42 | [0.84–2.38] | 7.9 | 0.85 | [0.51–1.41] |
| … to get ill | | | | | | | | | |
| No | NA | NA | NA | 7.4 | | | 9.4 | | |
| Yes | NA | NA | NA | 8.9 | 1.22 | [0.75–1.98] | 6.9 | 0.71 | [0.45–1.12] |
| … about longtime restrictions of social life | | | | | | | | | |
| No | NA | NA | NA | 7.5 | | | 8.7 | | |
| Yes | NA | NA | NA | 8.2 | 1.11 | [0.70–1.76] | 8.0 | 0.91 | [0.56–1.49] |
| ... to lose employment | | | | | | | | | |
| No | 9.9 | | | 8.0 | | | 9.2 | | |
| Yes | 11.8 | 1.03 | [0.65–1.65] | 6.9 | 0.85 | [0.47–1.54] | 5.5 | 0.57 | [0.32–1.03] |

* $p < 0.05$; ** $p < 0.01$, *** $p < 0.001$; marked in bold. Abbreviations: AU: alcohol use, DHS: German Head Office for Addiction Issues, OR: odds ratio, CI: confidence interval.
